# Acute immobilization stress following contextual fear conditioning reduces fear memory: timing is essential

**DOI:** 10.1186/s12993-016-0092-1

**Published:** 2016-02-24

**Authors:** Akemi Uwaya, Hyunjin Lee, Jonghyuk Park, Hosung Lee, Junko Muto, Sanae Nakajima, Shigeo Ohta, Toshio Mikami

**Affiliations:** Department of Biochemistry and Cell Biology, Institute for Advanced Medical Sciences, Nippon Medical School, 1-396 Kosugi-cho, Nakahara-ku, Kawasaki, Kanagawa 211-8533 Japan; Department of Laboratory Medicine, The Jikei University School of Medicine, 3-25-8, Nishi-Shimbashi, Minato-ku, Tokyo, 105-8641 Japan; Department of Cell Biology and Neuroscience, Juntendo Medical School, 2-1, Hongo, Bunkyo-ku, Tokyo, 113-8421 Japan; Graduate School of Health and Sport Science, Nippon Sport Science University, 7-1-1 Fukasawa, Setagaya-ku, Tokyo, 158-8508 Japan; Kyoritsu Women’s Junior College, 2-2-1 Hitotsubashi, Chiyoda-ku, Tokyo, 101-8437 Japan; Department of Health and Sport Science, Nippon Medical School, 1-7-1, Sakaiminami machi, Mushasino-shi, Tokyo 180-0023 Japan

**Keywords:** Histone acetylation, Fear conditioning, Stress, Corticosterone

## Abstract

**Background:**

Histone acetylation is regulated in response to stress and plays an important role in learning and memory. Chronic stress is known to deteriorate cognition, whereas acute stress facilitates memory formation. However, whether acute stress facilitates memory formation when it is applied after fear stimulation is not yet known. Therefore, this study aimed to investigate the effect of acute stress applied after fear training on memory formation, mRNA expression of brain-derived neurotrophic factor (BDNF), epigenetic regulation of BDNF expression, and corticosterone level in mice in vivo.

**Methods:**

Mice were subjected to acute immobilization stress for 30 min at 60 or 90 min after contextual fear conditioning training, and acetylation of histone 3 at lysine 14 (H3K14) and level of corticosterone were measured using western blot analysis and enzyme-linked immunosorbent assay (ELISA), respectively. A freezing behavior test was performed 24 h after training, and mRNA expression of BDNF was measured using real-time polymerase chain reactions. Different groups of mice were used for each test.

**Results:**

Freezing behavior significantly decreased with the down-regulation of BDNF mRNA expression caused by acute immobilization stress at 60 min after fear conditioning training owing to the reduction of H3K14 acetylation. However, BDNF mRNA expression and H3K14 acetylation were not reduced in animals subjected to immobilization stress at 90 min after the training. Further, the corticosterone level was significantly high in mice subjected to immobilization stress at 60 min after the training.

**Conclusion:**

Acute immobilization stress for 30 min at 60 min after fear conditioning training impaired memory formation and reduced BDNF mRNA expression and H3K14 acetylation in the hippocampus of mice owing to the high level of corticosterone.

**Electronic supplementary material:**

The online version of this article (doi:10.1186/s12993-016-0092-1) contains supplementary material, which is available to authorized users.

## Background

Adrenal glands release corticosterone in response to stress, and this hormone plays an important role in memory formation [1, 2]. It triggers the transcription of brain-derived neurotrophic factor (BDNF) via the activation of the *N*-methyl-d-aspartic acid (NMDA) receptor.

The contextual fear conditioning test is a well-established paradigm to investigate the neural mechanisms of learning and memory [3, 4]. Animals subjected to fear training showed enhanced expression of BDNF and an increase in freezing time after 24 h [5]; however, animals with impaired BDNF function due to *BDNF* gene knockout [6, 7] or treatment with an anti-BDNF neutralizing antibody [8] showed deteriorated memory, indicating that BDNF is critical for memory formation.

During memory formation, BDNF expression is mostly regulated by epigenetic modification, especially histone acetylation. Histone acetylation is associated with the regulation of the transcription of genes encoding proteins related to memory formation, thereby improving it [9–12]. For example, contextual fear training increases histone acetylation [3] and BDNF promoter binding to acetylated histone [12], followed by the consolidation of memory [12]; further, inhibition of histone deacetylation facilitates memory formation [9], indicating that the regulation of BDNF via histone acetylation plays an important role in memory formation [3, 13].

Chronic stress deteriorates cognition, and stress-induced deficits of cognition are attributed to epigenetic modifications such as increase in histone deacetylation and methylation [14]. On the other hand, when animals are subjected to acute stress, memory formation improves. That is, in the contextual fear conditioning test, acute stress applied before fear training facilitates memory [15]. Further, histone acetylation in the hippocampus regulates memory formation [16]. However, whether acute stress impairs or facilitates memory formation when stress is applied after fear training is not yet known. This study aimed to investigate the effect of acute stress applied after fear training on contextual fear memory formation in mice. To this end, we selected a contextual fear memory paradigm and histone acetylation marks because memory formation induced by contextual fear conditioning training requires histone acetylation, which occurs during a short period following such training [3].

## Methods

### Animals

All experimental procedures and animal treatments were performed in accordance with the laboratory animal manual guidelines of Nippon Medical School. This study was approved by the Animal Care and Use Committee of Nippon Medical School (Tokyo, Japan) and the approval number was 24-029. Male C57/BL6 mice (Sankyo Lab Service, Japan), aged 10 weeks and weighing 24.1 ± 0.75 g, were used. These animals were housed under a 12-h light/dark schedule and given access to rodent chow (Oriental Yeast Co., Japan) and water ad libitum.

### Experimental Protocol

We performed six experiments. Each experiment was designed and performed based upon the results of previous experiments and used a separate group of mice.

### Experiment 1: contextual fear conditioning training

**Fig. 1:**
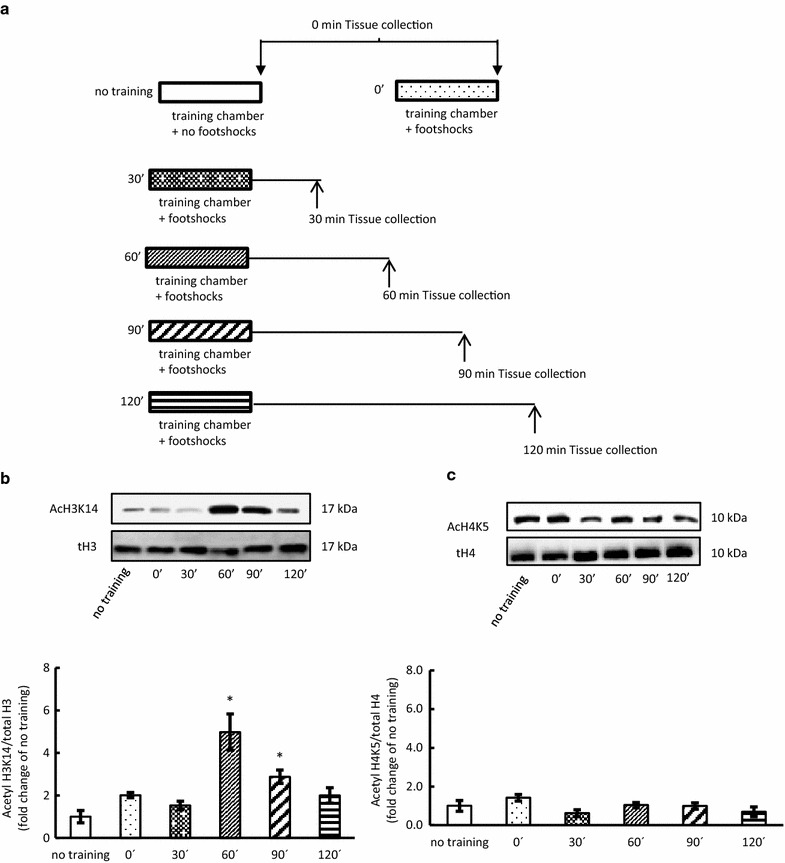
Effect of contextual fear conditioning (Experiment 1). **a** Experimental protocol for contextual fear conditioning training. Hippocampus samples were collected at 0, 30, 60, 90, 120 min after contextual fear conditioning training. **b** Representative western blots for the acetylation of H3K14 in the hippocampus and quantification of immunoblot densities for mean (±SEM) acetylated H3K14 at each time point after the contextual fear conditioning training compared with that in mice without training (n = 3–4). **c** Representative western blots for acetylation of H4K5 in the hippocampus and quantification of immunoblot densities for mean (±SEM) acetylated H4K5 at each time point after the contextual fear conditioning training compared with that in mice without training (n = 3–4). Data are expressed as mean ± SEM. *P < 0.05 compared with no training

Mice were randomly divided into six groups: no training, 0, 30, 60, 90, and 120 min. The mice were sacrificed to collect hippocampus samples at 0, 30, 60, 90, or 120 min after contextual fear conditioning training to examine acetylated H3K14 and acetylated H4K5 (Fig. 1a). The no training mice were allowed to explore the training chamber, but did not receive any foot shock. The hippocampus samples of the no training mice were collected immediately after removal from the contextual fear conditioning chamber.

Contextual fear conditioning was applied according to published protocols with slight modifications [17]. The mice were transported to an animal experimental laboratory and allowed to acclimate for at least 30 min prior to contextual fear conditioning training. The mice were then placed in the foot shock system model MK-450MSQ (Muromachi Kikai CO. LTD, Japan) and allowed to explore for 2 min followed by three electric foot shocks (0.8 mA, 2-s duration and 2-min interval). Animals were left in the apparatus for a further minute before being removed.

Histone extraction was performed according to published protocols with slight modifications [17]. For histone extraction experiments, animals were sacrificed, and the brains were removed and hippocampi were dissected. Hippocampus samples were homogenized for 10 strokes in homogenizing buffer [250 mM sucrose (Wako, Japan), 50 mM Tris–HCl pH 7.5, 25 mM KCl (Kato Chemical Co., Inc., Japan), 0.5 mM phenylmethylsulfonyl fluoride (PMSF; Sigma, USA), 0.9 mM Na t-butyrate (Sigma), 1 % protein inhibitor cocktail (Sigma)] using a Dounce homogenizer. All steps were performed on ice, and all centrifugations were performed at 4 °C. Homogenized samples were centrifuged at 770×*g* for 1 min. The supernatant was removed and then re-suspended in 0.5 mL of 0.4 N H2SO4 for 30 min to extract histones. Samples were centrifuged at 14,000×*g* for 10 min. The supernatant was transferred to a fresh tube, and 250 μL of trichloroacetic acid (with 4 mg/mL deoxycholic acid) was added to the precipitated proteins. The precipitate was incubated on ice for 30 min and then centrifuged at 14,000×*g* for 30 min. The supernatant was discarded and the protein pellet was washed with 1 mL of acidified acetone (0.1 % HCl) and 1 mL of pure acetone for 5 min each, with centrifugation at 14,000×*g* for 5 min after each wash. The final protein pellet was resuspended in 10 mM Tris–HCl pH 8.0 and stored at −80 °C.

### Experiment 2: measurement of freezing time and measuring mRNA levels by RT-PCR

**Fig. 2:**
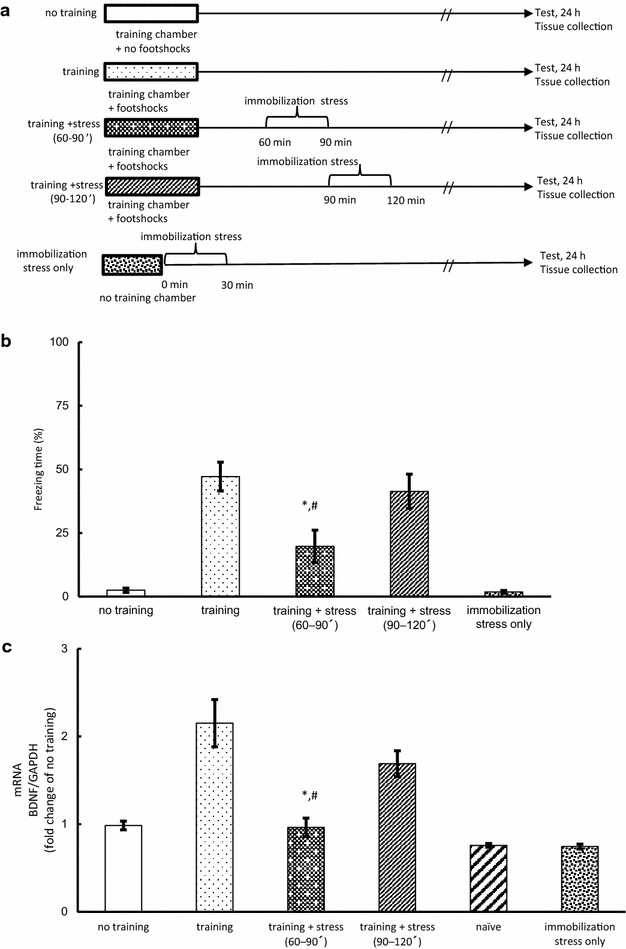
Effect of acute immobilization stress after contextual fear conditioning (Experiment 2). **a** Experimental protocol for contextual fear conditioning training followed by stress and behavior tests. **b** Quantification of freezing behavior 24 h after the contextual fear conditioning training followed by immobilization stress (n = 10). **c** Quantification of BDNF/GAPDH mRNA ratio in the hippocampus (n = 5–8). The samples of naïve mice were collected the same day as the other groups. Naïve mice were not exposed to the training apparatus or immobilization stress. Data are expressed as mean ± SEM. *P < 0.05 compared with training group. ^#^ *P* < 0.05 compared with training + stress group (90–120 min)

Mice were randomly divided into six groups: no training, training, training + stress (60–90 min), training + stress (90–120 min), immobilization stress only, and naïve. Naïve mice were kept in their home cage until sample collection. The no training mice were exposed to the training chamber without footshock, and then returned to their home cage. Training mice were returned to their home cage immediately after contextual fear conditioning training. After contextual fear conditioning training, training + stress (60–90 min), and training + stress (90–120 min) mice were subjected to immobilization stress for 30 min, and then returned to their home cage. Immobilization stress only mice were subjected to immobilization stress for 30 min, and then returned to their home cage. Twenty-four hours after the training, all mice were subjected to the measurement of freezing time and sacrificed to collect hippocampus samples. The hippocampus samples were used for analyzing mRNA (Fig. 2a).

Immobilization stress was applied according to published protocols with slight modifications [18]. Mice were exposed to 30 min of immobilization in an immobilization cage (width: 3 cm, length: 3 cm, height: 7.5 cm) 60 or 90 min after contextual fear conditioning training.

Freezing behavior (defined as complete lack of movement, except for respiration) was measured by observing the animals for 3 min, 24 h after training. The absence of all non-respiratory movement was measured and hand-scored by trained observers. Memory was assessed as the percentage of time that the mice spent freezing when placed back in the training apparatus without receiving footshocks.

The mice were sacrificed by decapitation immediately after the fear conditioning behavioral test, and the hippocampi were isolated. Hippocampal BDNF mRNA (NCBI accession no. NM-007540.4) was measured. Total RNA was isolated using the RNeasy Mini Kit (QIAGEN, Germany) according to the manufacturer’s instructions. RNA quantification was carried out by measuring absorption at 260 nm. Complementary DNA was generated from total RNA by reverse transcription (RT) using oligo(dT) [19] 12–18 primers (Invitrogen, USA) and superscript reverse transcriptase (Invitrogen, USA) in PCR thermal cycler DICE (Takara, Japan). The RT steps consisted of incubation at 37 °C for 10 min followed by incubation at 50 °C for 60 min. BDNF mRNA level in the hippocampus was measured by real-time quantitative PCR. Glyceraldehyde-3-phosphate dehydrogenase (GAPDH) served as an endogenous control. Quantification of the TaqMan^®^ real-time PCR results was performed by plotting fluorescent signal intensity against the number of PCR cycles on a semi-logarithmic scale. The fluorescent probes and the forward and reverse primers were designed using Primer 3 software based on information from NCBI accession no. NM-007540.4 and synthesized by Hokkaido System Science (Hokkaido System Science Co., Japan). The primer and probe sequences were as follows: BDNF probe: 5ʹ-ACACTTCCCGGGTGATGCTCAGCA-3ʹ, BDNF reverse primer: 5ʹ-GAGGCTCCAAAGGCACTTGA-3ʹ, BDNF forward primer: 5ʹ-ACCATAAGGACGCGGACTTG-3ʹ, GAPDH probe: 5ʹ-TGGATGGCCCCTCTGGAAAGCTG-3ʹ, GAPDH reverse primer: 5ʹ-ATGTTCTGGGCAGCC-3ʹ, and GAPDH forward primer: 5ʹ-CATCACTGCCACCCAGAAGA-3ʹ. The reaction protocol for real-time PCR consisted of 50 °C for 5 min followed by 95 °C for 5 min. This was followed by 40 cycles of a two-step PCR reaction consisting of 95 °C for 20 s and 60 °C for 1 min. The real-time PCR values for BDNF were corrected relative to the values for GAPDH.

### Experiment 3: western blotting

**Fig. 3:**
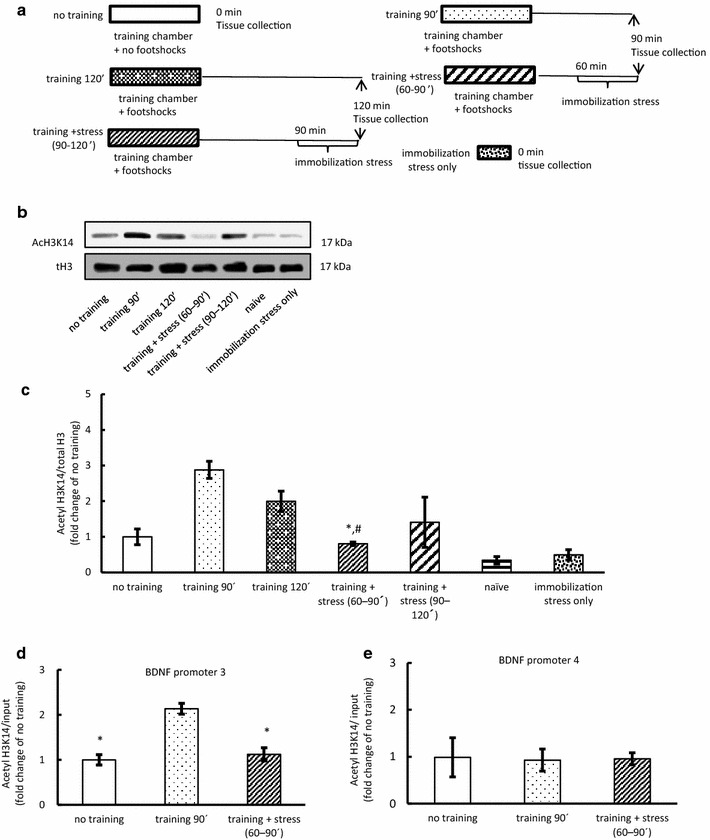
Epigenetic modification of BDNF promoter via acute immobilization stress applied after fear training (Experiments 3 and 4). **a** Experimental protocol for contextual fear conditioning training followed by immobilization stress. **b** Representative western blots for the acetylation of H3K14 in the hippocampus at 90 and 120 min after the training and quantification of immunoblot densities for mean (± SEM) acetylated H3K14 at 90 and 120 min after the training compared with that in the without training group (n = 4–5). **c** Acetylated H3K14 levels in the hippocampus at *bdnf* promoter 3 at 90 min after the training compared with that in the no training group (n = 3–4). **d** Levels of H3K14 acetylation in the hippocampus at *bdnf* promoter 3 at 90 min after the training compared with that in the group without training (n = 3–4). **e** Levels of H3K14 acetylation in the hippocampus at *bdnf* promoter 4 at 90 min after the training compared with that in the group without training (n = 4). Data are expressed as mean ± SEM. *P < 0.05 compared with training 90 min. ^#^ *P* < 0.05 compared with training 120 min. Different sets of mice were used for western blotting and ChIP assay analyses

Mice were randomly divided into seven groups: no training, training 90 min, training 120 min, training + stress (60–90 min), training + stress (90–120 min), immobilization stress only, and naïve. Naïve mice remained in their home cage until sample collection. The no training mice were sacrificed to collect hippocampus samples immediately after exposure to the training apparatus without footshock. Training 90 min and training 120 min mice were sacrificed to collect the hippocampus, 90 and 120 min after the training respectively. Training + stress (60–90 min) and training + stress (90–120 min) mice were sacrificed to collect the hippocampus immediately after the immobilization stress following training. The hippocampus samples were used for western blotting (Fig. 3a).

Extracted histone protein concentrations were measured using commercially available reagents (BCATM Protein Assay Reagents, Pierce, USA). Four volumes of sample buffer (final concentration 6.25 mM Tris–HCl, pH 6.8, 2 % sodium dodecyl sulfate (SDS, Wako), 10 % glycerol (Wako), 1.25 % 2-mercaptoethanol (Wako), 0.1 % bromophenol blue (Wako) was added to each sample. One microgram of protein for acetylation of H3K14 and H4K5, and 0.5 microgram of protein for total H3 and total H4 from each sample was loaded and run on a 4 % acrylamide stacking gel and 15 % acrylamide resolving gel. Proteins were transferred to polyvinylidene difluoride membranes, which were processed for immunoblotting. These membranes were first blocked in 3 % bovine serum albumin in Tris-buffered saline with Tween (TTBS) (150 mM NaCl, 20 mM Tris–HCl pH 7.5, 0.05 % Tween-20) for 60 min at room temperature and then incubated with primary antibody overnight at 4 °C. The primary antibodies and dilutions used were anti-histone H3 (1:500, Millipore), anti-acetyl-histone H3 (Lys14, 1:1000, Millipore), anti-histone H4 (1:500, Millipore), and anti-acetyl-histone H4 (Lys5, 1:1000, Millipore). Subsequently, membranes were washed with TTBS and incubated with secondary antibody for 2.5 h at room temperature. The secondary antibody used was anti-rabbit IgG, horseradish peroxidase-linked antibody (1:3000, Cell Signaling). Finally, membranes were washed with TTBS and then immunolabeled using chemical luminescence with Immunostar LD (Wako, Tokyo). The luminescence was detected with an LAS 1000 mini-image analyzer (Fuji Film, Tokyo). Densitometric analysis was performed using Image Gauge ver. 4.0 (Fuji Film).

### Experiment 4: chromatin immunoprecipitation (ChIP)

Mice were randomly divided into three groups: no training, training 90 min, and training + stress (60–90 min). Immediately after exposure to the training chamber without footshock, the no training mice were sacrificed to collect the hippocampus samples. Training 90 min mice were sacrificed to collect the hippocampus 90 min after the training. Training + stress (60–90 min) mice were sacrificed to collect the hippocampus immediately after the immobilization stress following training. The hippocampus samples were used for a chromatin immunoprecipitation (ChIP) assay.

ChIP was performed following a modified version of the Millipore ChIP kit protocol. Immediately after hippocampus tissue disruption, the sample was cross-linked in formalin for 15 min at room temperature. The crosslinking reaction was stopped by adding glycine at a final concentration of 0.125 M. The tissue was washed with cold PBS containing 1 mM PMSF and a protease inhibitor cocktail (Sigma). Then, the sample was homogenized in cell lysis buffer (10 mM Tris–HCl pH 8.0, 10 mM NaCl, 0.2 % NP-40, 1 mM PMSF and protease inhibitors cocktail) for 10 strokes using a Dounce homogenizer. The homogenized sample was centrifuged at 4000×*g* for 5 min at 4 °C, and the supernatant was removed. Nuclear lysis buffer (1 % SDS, 10 mM EDTA, 50 mM Tris–HCl pH 8.0, 1 mM PMSF and protease inhibitors cocktail) was added to the precipitate and incubated for 10 min on ice, followed by sonication using Bioruptor^®^ (Cosmo Bio Co., Ltd.). Next, DNA fragments were centrifuged at 10,000×*g* for 15 min at 4 °C. Twenty microliters of lysate was saved as “input” for later normalization. The remaining lysate was diluted with ChIP dilution buffer (1.1 % Triton X-100, 0.01 % SDS, 1.2 mM EDTA, 167 mM NaCl, 16.7 mM Tris–HCl pH 8.0, 1 mM PMSF and protease inhibitor cocktail) at a 1:10 ratio. The chromatin solution was pre-cleared with 75 μL of protein A agarose/salmon sperm DNA (50 % slurry, Millipore) for 1 h at 4 °C and centrifuged at 3000×*g* for 5 min at 4 °C, with the supernatant recovered. This supernatant was immunoprecipitated overnight at 4 °C with 3 μL of antibody (H3K14, Millipore). After immunoprecipitation, the DNA-histone complex was collected with 60 μL of protein A agarose/salmon sperm DNA (50 % slurry) for 1 h at 4 °C, followed by one wash in low-salt buffer (0.1 % SDS, 1 % Triton X-100, 2 mM EDTA, 20 mM Tris–HCl pH 8.0, 150 mM NaCl), one wash in high-salt buffer (0.1 % SDS, 1 % Triton X-100, 2 mM EDTA, 20 mM Tris–HCl pH 8.0, 500 mM NaCl), one wash in LiCl buffer (0.25 M LiCl, 1 % NP-40, 1 % deoxycholate Na, 1 mM EDTA, 10 mM Tris–HCl pH 8.0) and two washes in Tris–EDTA buffer pH 8.0 (Wako). The precipitated protein-DNA complexes were eluted from the antibody with elution buffer (1 % SDS, 50 mM NaHCO_3_). The elution buffer was added to the input, and then incubated overnight at 65 °C in 200 mM NaCl to reverse the formaldehyde cross-links. Ten microliters of 0.5 M EDTA, 20 μL of 1 M Tris–HCl pH 6.5 and 2 μL of 10 mg/mL proteinase K (Sigma) were added to the elutes and incubated for 1 h at 45 °C. DNA was extracted using phenol/chloroform/isoamyl alcohol and then precipitated with ethanol. Next, quantitative PCR was performed with primers specific to the *bdnf* gene promoters. Specific primers were designed to amplify proximal promoter regions and used as described previously [14]. For *bdnf* P3, the forward primer was 5′-GTGAGAACCTGGGGCAAATC-3′ and the reverse primer was 5′-ACGGAAAAGAGGGAGGGAAA-3′. For *bdnf* P4, the forward primer was 5′-CTTCTGTGTGCGTGAATTTGCT-3′ and the reverse primer was 5′-AGTCCACGAGAGGGCTCCA-3′. Input and immunoprecipitated DNA amplification reactions were run in the presence of SYBR Green (real-time PCR master mix). The cumulative fluorescence for each amplification was normalized to the input amplification.

### Experiment 5: measurement of plasma corticosterone

**Fig. 4:**
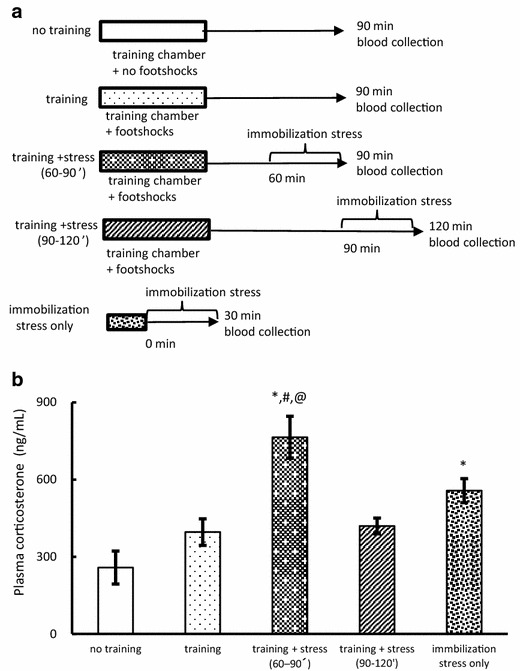
Plasma corticosterone levels (Experiment 5). **a** Experimental protocol for blood sample collection. **b** Comparison of plasma corticosterone levels (n = 5). Data are expressed as mean ± SEM. *P < 0.05 compared with no training group. ^#^ *P* < 0.05 compared with training group. ^@^ *P* < 0.05 compared with training + stress group (90–120 min)

Mice were randomly divided into five groups: no training, training 90 min, training + stress (60–90 min), training + stress (90–120 min), and immobilization stress only. Ninety minutes after exposure the training chamber without footshock, the no training mice were sacrificed to collect the blood samples. Training 90 min mice were sacrificed to collect the blood 90 min after the training. Training + stress (60–90 min) and training + stress (90–120 min) mice were sacrificed to collect the blood immediately after the immobilization stress following training. Immobilization stress only mice were sacrificed to collect the blood sample immediately after the immobilization stress. The blood samples were used for corticosterone measurement (Fig. 4a).

Blood samples were collected after contextual fear training and immobilization stress. Plasma corticosterone levels were quantified by ELISA according to the instructions of the supplier (AssayMax Corticosterone ELISA Kit, AssayPro LLC, USA).

### Experiment 6: effect of glucocorticoid receptor antagonist (mifeprostone) injection after contextual fear conditioning training

**Fig. 5:**
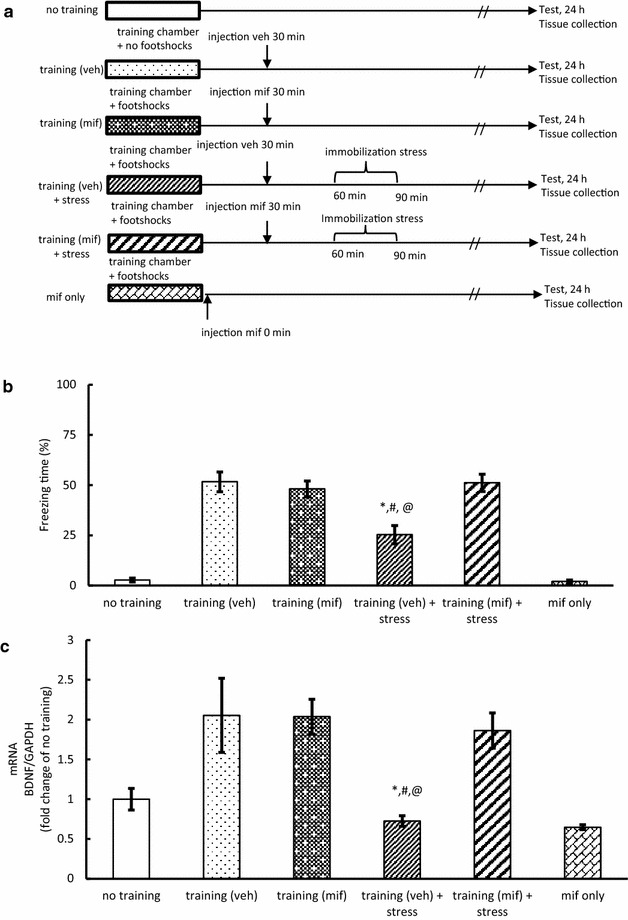
Effect of glucocorticoid receptor antagonist (mifepristone) injection after contextual fear conditioning training (Experiment 6). **a** Experimental protocol for contextual fear conditioning training followed by stress and behavior tests **b** quantification of freezing behavior 24 h after the contextual fear conditioning training followed by immobilization stress (n = 9–11). **c** Quantification of BDNF/GAPDH mRNA ratio in the hippocampus (n = 6–8). *P < 0.05 compared with training (veh). ^#^ *P* < 0.05 compared with training (mif). ^@^P < 0.05 compared with training (mif) + stress

The glucocorticoid receptor antagonist mifepristone (mif, Sigma, 10 mg/kg) or vehicle (veh., propylene glycol, Wako) was injected subcutaneously 30 min after contextual fear conditioning training [20]. The mice were randomly divided into six groups: no training, training (veh), training (mif), training (veh) + stress, training (mif) + stress, and mif only. After exposure to the training chamber without footshock, the no training mice were returned to their home cage. Immediately after the injection of mifepristone (mif), mif only mice were returned to their home cage. Thirty min after contextual fear conditioning training, the other groups of mice were injected with veh or mif as appropriate. Immediately after the injection, training (veh) and training (mif) mice were returned to their home cage. Training + stress (60–90 min) and training + stress (90–120 min) mice were subjected to immobilization stress for 30 min, then returned to their home cage. Twenty-four hours after training, all mice were subjected to the measurement of freezing time and sacrificed to collect hippocampus samples. The hippocampus samples were used for analyzing mRNA BDNF as in Experiment 2 (Fig. 5a).

### Statistical analysis

All values are shown as the mean ± standard error of measurement (SEM). One-way analysis of variance (ANOVA), followed by the Tukey post hoc test was used for comparisons between groups. Statistical significance was accepted at *P* < 0.05. SPSS 21 software was used to perform the statistical analysis. Additional file 1: Table S1, Additional file 2: Table S2, Additional file 3: Table S3, Additional file 4: Table S4, Additional file 5: Table S5, Additional file 6: Table S6, Additional file 7: Table S7, Additional file 8: Table S8, Additional file 9: Table S9, Additional file 10: Table S10 show details of the Tukey post hoc tests.

## Results

### Time course of histone acetylation in the hippocampus (Experiment 1)

Memory formation induced by contextual fear conditioning training requires histone acetylation that occurs during a short period following such training [3]. To investigate the critical timing for histone acetylation following contextual fear training, we examined the time course change in histone acetylation after such training. Acetylation of H3 at lysine 14 (H3K14) in the hippocampus was significantly increased at 60 and 90 min after fear training (F_(5,17)_ = 12.33, *P* < 0.05; Fig. 1b, Additional file 1: Table S1). However, acetylation of H4 at lysine 5 (H4K5) did not change at any time point (Fig. 1c, Additional file 2: Table S2). These findings indicate that acetylation of H3K14, but not H4K5, is involved in memory formation, and that the critical timing for histone acetylation is from 60 to 90 min after the training.

### Effect of acute immobilization stress after contextual fear conditioning (Experiment 2)

Based on our finding that H3K14 acetylation was enhanced from 60 to 90 min after the training, we examined whether freezing behavior was influenced when acute stress was applied while histone acetylation was elevated. For this purpose, the trained mice were subjected to 30 min of acute immobilization 60 or 90 min after fear training and then subjected to the measurement of freezing time 24 h later. Mice in the immobilization stress only group were subjected to immobilization stress for 30 min, without fear training beforehand. Immobilization at 60 min after fear training significantly reduced the freezing time as compared to that when the mice were subjected to training alone (F_(4,45)_ = 78.73, *P* < 0.05; Fig. 2b, Additional file 3: Table S3); however, immobilization applied at 90 min after fear training did not reduce the freezing time (Fig. 2b).

BDNF expression is critically involved in the consolidation phase of long-term memory [5]. Therefore, we analyzed the expression of BDNF mRNA. Hippocampus samples were collected immediately after the measurement of freezing time. Naïve mice did not explore the training apparatus, nor were they subjected to immobilization stress. Training and training + stress (90–120 min) mice showed significantly increased expression of BDNF mRNA compared with those without training (i.e., naïve, no training, and immobilization stress only mice), whereas mice in the training + stress (60–90 min) group did not show increased expression of BDNF mRNA (F_(5,34)_ = 12.75, *P* < 0.05; Fig. 2c, Additional file 4: Table S4). Taken together, the results indicate that immobilization stress affected expression of BDNF mRNA at 60–90 min, but not 90–120 min after fear training.

### Epigenetic modification of BDNF promoter in response to acute stress applied after fear training (Experiment 3 and 4)

Based on the above findings, we examined whether immobilization following fear training influences epigenetic change via histone acetylation in BDNF transcription. At 90 and 120 min after fear training, a significant increase in the acetylation of H3K14 was noted compared with that in the groups without training. No significant difference was observed between training + stress (60–90 min) and training + stress (90–120 min) (*P* = 0.228); however, training + stress (60–90 min), but not training + stress (90–120 min), significantly reduced acetylation (F_(6,26)_ = 19.47, *P* < 0.05; Fig. 3c, Additional file 5: Table S5). These data suggested that around 60 min after fear training is a critical time for memory formation. Next, we analyzed H3K14 acetylated at BDNF promoters 3 and 4 in the hippocampus. Acetylation of H3K14 at promoter 3, but not at promoter 4, after training for 90 min was significantly elevated (F_(2,8)_ = 22.86, *P* < 0.05; Fig. 3d, Additional file 6: Table S6, Additional file 7: Table S7). In addition, training + stress (60–90ʹ) reduced the acetylation of H3K14 at promoter 3 compared to that in the no training group (F_(2,8)_ = 22.86, *P* < 0.05; Fig. 3d). These results suggest that immobilization applied at 60 min after fear training might inhibit memory formation by suppressing the acetylation of H3K14 at BDNF promoter 3 in the hippocampus.

### Effect of glucocorticoid on memory formation and BDNF expression (Experiment 5 and 6)

On the basis of the findings, we hypothesized that an increase in corticosterone due to immobilization would be the primary cause of a disturbance of memory formation, since corticosterone is one of the hormones regulating memory [21]. To evaluate our hypothesis, we measured plasma corticosterone levels 90 and 120 min after training. Mice in the training + stress (60–90 min) group showed a remarkable increase in the plasma corticosterone level as compared to that in the training, training + stress (90–120 min), and no training (F_(4,20)_ = 11.05, *P* < 0.05; Fig. 4b, Additional file 8: Table S8) mice. A significant difference was observed between the no training and immobilization stress only groups. However, no significant difference was noted among the training, training + stress (90–120 min), and immobilization stress only groups. The high level of corticosterone of training + stress (60–90 min) was probably due to the synergistic effect of training and immobilization stress from 60 to 90 min post training, which might block memory formation following contextual fear training.

This was confirmed by injecting the mice with mifepristone (mif), a glucocorticoid receptor antagonist, or vehicle (veh) at 30 min before immobilization stress (Experiment 6). Mice were subjected to 30 min of immobilization at 60 min after the training, and the freezing time was measured after 24 h (Fig. 5b). The freezing time significantly decreased in the mice in the training (veh) + stress group as compared to the trained mice without immobilization stress [i.e., training (veh) and training (mif)], whereas training (mif) + stress reverted the freezing time to that of the training (veh) mice (F_(5,55)_ = 40.29, *P* < 0.05; Fig. 5b, Additional file 9: Table S9). In addition, training (veh) + stress led to the down-regulation of BDNF mRNA, and training (mif) + stress reverted the BDNF mRNA expression level to that of the training (veh) and the training (mif) levels (F_(5,34)_ = 7.89, *P* < 0.05; Fig. 5c, Additional file 10: Table S10). These findings indicate that the high level of corticosterone induced in response to acute stress impairs memory formation.

## Discussion

Chronic stress is known to deteriorate cognition [14], whereas acute stress is known to have a positive effect on memory formation [15]. However, whether acute stress facilitates memory formation when stress is applied after fear training has not yet been determined. Our study showed that acute immobilization stress applied at 60 min but not 90 min post-training could affect freezing time (Fig. 2b, c), enabling us to speculate that the interference during the memory consolidation (60–90 min post-training) could impair memory formation, but that the interference following the memory consolidation (90–120 min post-training) could have no effect on the memory formation.

Several studies have shown that BDNF contributes to memory formation. For example, knockdown of hippocampal BDNF expression was shown to decrease the freezing behavior in the contextual fear conditioning test [7]. Inhibition of BDNF function by using neutralizing antibody against BDNF led to the formation of spatial memory alone [8]. In this study, trained mice showed an increase in freezing behavior and hippocampal BDNF mRNA expression, whereas they were decreased in mice subjected to immobilization at 60 min after the training (Fig. 2b, c), indicating that around 60 min after fear training is critical for memory formation in mice.

BDNF transcription after fear training is epigenetically regulated, especially by histone acetylation [3]. There are many lysine residues on the N-terminal tails of histone H3 and H4; for this study we selected lysine 14 for histone H3 and lysine 5 for histone H4 because the sites are the closest N-terminal tails and acetylation specific [22], and acetylation of the sites increases in the hippocampus following contextual fear conditioning for the establishment of contextual fear memory [4]. Our data showed that fear training increases the acetylation of H3K14, but not of H4K5 (Fig. 1b, c) in the hippocampus; this result is consistent with those of previous studies [3, 9, 13]. In addition, 30 min of immobilization stress at 60 min after fear training, but not immobilization stress at 90 min, reduced the acetylation (Fig. 3b). Our chromatin chip assay showed that the binding of *bdnf* promoter 3 to H3K14 was increased following fear training and suppressed by the immobilization stress; however, no changes were noted in the function of promoter 4 (Fig. 3c, d). These findings suggest that fear memory formation might be mediated by the regulation of BDNF transcription via the acetylation of H3K14 at *bdnf* promoter 3. However, other epigenetic modifications are also required for memory formation. Gupta et al. indicated that histone methylation, especially trimethylation of histone H3 at lysine 4, is required for the accurate long-term consolidation of contextual fear memories [23]. Chwang et al. showed that histone H3 phosphorylation in hippocampal area CA1 is regulated after a behavioral fear-conditioning paradigm [17]. These findings indicate that, besides acetylation, other types of histone modifications (phosphorylation, methylation, etc.) need to be investigated following acute stress. Bredy et al. demonstrated that fear conditioning and extinction result in distinct patterns of histone acetylation of H3 and H4, and suggested that acetylated H3 increased after fear training, and that acetylated H4 increased after extinction training [24]. This study supported our finding that acetylated histone H4 was not involved BDNF gene expression in fear condition paradigm (Fig. 1c).

Chronic and acute actions of glucocorticoids on memory processes differ in many respects, including differences in behavior and the molecular mechanisms. In general, an increase in corticosterone due to chronic stress can be a cause of disturbance in memory formation [25]. In this study, acute immobilization at 60 min after fear conditioning training significantly increased plasma corticosterone levels as compared to those in trained mice (Fig. 4b). Furthermore, both freezing time and BDNF mRNA expression level of mice subjected to immobilization at 60 min were ameliorated by injecting a glucocorticoid receptor antagonist, mifepristone, 30 min before immobilization (Fig. 5b, c). However, the corticosterone level of mice subjected to immobilization at 90 min after the training reverted to the level in trained mice. These findings indicate that the impediment of memory formation by acute immobilization stress applied at 60 min after training can be attributed to a high level of corticosterone.

A possible mechanism of memory formation is that glucocorticoid released by fear training enhances the activation of NMDA receptors via glutamate and the transcription of BDNF by extracellular signal-regulated kinase [3]. Thus, glucocorticoid triggers the transcription of BDNF, resulting in enhanced memory. However, acute stress applied at 60 min after fear training might further increase the release of corticosterone from the adrenal cortex. When acute immobilization stress was applied at 60 min after fear training, the additional increase in corticosterone might have disrupted epigenetic modification associated with BDNF transcription, followed by the impairment of memory formation. Abrari et al. suggested that the administration of corticosterone after memory training enhanced memory formation in a dose-dependent manner, but excess administration of corticosterone reduced freezing behavior to the level noted in the vehicle control group [26]. These and our findings confirm that learning and synapse plasticity depend on the level of corticosterone: when its level is very low or high, memory formation is impaired, whereas an intermediate level of corticosterone facilitates memory formation [21].

To our knowledge, this is the first study to show that acute immobilization stress after contextual fear conditioning affects memory formation, including freezing behavior, BDNF mRNA expression, and acetylation of H3K14 and BDNF promoter 3 in the hippocampus. Owing to the high level of corticosterone, acute immobilization stress applied at 60 min after contextual fear training had an adverse effect, but not at 90 min after training. Facilitation or impairment of fearful memory formation depends on not only the timing of stress stimulation but also the level of corticosterone. This finding might be useful for memory study, including investigation of the mechanism underlying memory formation.

## Conclusions

In this study, we investigated the effect of acute stress applied after fear training on memory formation, BDNF expression, epigenetic regulation of BDNF expression, and corticosterone level. Our results showed that 60 min after contextual fear training is the critical time for memory formation in mice. When acute immobilization stress was applied during this critical time, freezing behavior significantly decreased, along with reduction in BDNF mRNA expression and H3K14 acetylation in the hippocampus, owing to the high level of corticosterone.

## Additional files

10.1186/s12993-016-0092-1 Tukey HSD for acetylation of H3K14 (Experiment 1).
10.1186/s12993-016-0092-1 Tukey HSD for acetylation of H4K5 (Experiment 1).
10.1186/s12993-016-0092-1 Tukey HSD for behavioral test 1 (Experiment 2).
10.1186/s12993-016-0092-1 Tukey HSD for BDNF analysis 1 (Experiment 2).
10.1186/s12993-016-0092-1 Tukey HSD for acetylation H3K14 (Experiment 3).
10.1186/s12993-016-0092-1 Tukey HSD for acetylation H3K14 at promoter 3 (Experiment 4).
10.1186/s12993-016-0092-1 Tukey HSD for acetylation H3K14 at promoter 4 (Experiment 4).
10.1186/s12993-016-0092-1 Tukey HSD for corticosterone analysis (Experiment 5).
10.1186/s12993-016-0092-1 Tukey HSD for behavioral test 2 (Experiment 6).
10.1186/s12993-016-0092-1 Tukey HSD for mRNA 2 (Experiment 6).

## Abbreviations

H3K14: histone 3 at lysine 14
H4K5: histone 4 at lysine 5
ELISA: enzyme-linked immunosorbent assay
BDNF: brained-derived neurotrophic factor
RT-PCR: reverse transcription polymerase chain reaction
GAPDH: glyceraldehyde-3-phsphate dehydrogenase
NCBI: national center for biotechnology Information
MSF: phenylmethylsulfonyl fluoride
SDS: sodium dodecyl sulfate
TTBS: tris-buffered saline with Tween
ChIP: chromatin immunoprecipitation
PBS: phosphate buffered saline
mif: mifepristone
SEM: standard error of measurement
ANOVA: analysis of variance
NMDA: n-methyl-d-aspartic acid
