# Supplementary material for: Acute immobilization stress following contextual fear conditioning reduces fear memory: timing is essential
Source: Behav Brain Funct. 2016 Feb 24;12:8. doi: 10.1186/s12993-016-0092-1 (PMC4765063; doi:10.1186/s12993-016-0092-1)
Supplement: Supplementary file 1 — 10.1186/s12993-016-0092-1 Tukey HSD for acetylation of H3K14 (Experiment 1). [file 12993_2016_92_MOESM1_ESM.docx]

Additional file 1

Table S1. Tukey HSD for acetylation of H3K14 (Experiment 1)

|  | | | |  |  |  |
| --- | --- | --- | --- | --- | --- | --- |
|  |  | Mean difference (I-J) | Std.Error | Sig. | 95% Confidence Interval | |
| (I) Course | (J) Course |  |  |  | Lower Bound | Upper Bound |
| no training | 0' | -.97500 | .51419 | .437 | -2.6197 | .6697 |
|  | 30' | -.52500 | .51419 | .904 | -2.1697 | 1.1197 |
|  | 60' | -3.96667^*^ | .55538 | .000 | -5.7432 | -2.1902 |
|  | 90' | -1.87500^*^ | .51419 | .021 | -3.5197 | -.2303 |
|  | 120' | -1.00000 | .51419 | .411 | -2.6447 | .6447 |
| 0' | no training | .97500 | .51419 | .437 | -.6697 | 2.6197 |
|  | 30' | .45000 | .51419 | .947 | -1.1947 | 2.0947 |
|  | 60' | -2.99167^*^ | .55538 | .001 | -4.7682 | -1.2152 |
|  | 90' | -.90000 | .51419 | .520 | -2.5447 | .7447 |
|  | 120' | -.02500 | .51419 | 1.000 | -1.6697 | 1.6197 |
| 30' | no training | .52500 | .51419 | .904 | -1.1197 | 2.1697 |
|  | 0' | -.45000 | .51419 | .947 | -2.0947 | 1.1947 |
|  | 60' | -3.44167^*^ | .55538 | .000 | -5.2182 | -1.6652 |
|  | 90' | -1.35000 | .51419 | .144 | -2.9947 | .2947 |
|  | 120' | -.47500 | .51419 | .935 | -2.1197 | 1.1697 |
| 60' | no training | 3.96667^*^ | .55538 | .000 | 2.1902 | 5.7432 |
|  | 0' | 2.99167^*^ | .55538 | .001 | 1.2152 | 4.7682 |
|  | 30' | 3.44167^*^ | .55538 | .000 | 1.6652 | 5.2182 |
|  | 90' | 2.09167^*^ | .55538 | .016 | .3152 | 3.8682 |
|  | 120' | 2.96667^*^ | .55538 | .001 | 1.1902 | 4.7432 |
| 90' | no training | 1.87500^*^ | .51419 | .021 | .2303 | 3.5197 |
|  | 0' | .90000 | .51419 | .520 | -.7447 | 2.5447 |
|  | 30' | 1.35000 | .51419 | .144 | -.2947 | 2.9947 |
|  | 60' | -2.09167^*^ | .55538 | .016 | -3.8682 | -.3152 |
|  | 120' | .87500 | .51419 | .549 | -.7697 | 2.5197 |
| 120' | no training | 1.00000 | .51419 | .411 | -.6447 | 2.6447 |
|  | 0' | .02500 | .51419 | 1.000 | -1.6197 | 1.6697 |
|  | 30' | .47500 | .51419 | .935 | -1.1697 | 2.1197 |
|  | 60' | -2.96667^*^ | .55538 | .001 | -4.7432 | -1.1902 |
|  | 90' | -.87500 | .51419 | .549 | -2.5197 | .7697 |
| * The mean difference is significant at the 0.05 level. | | | | |  |  |
