# Supplementary material for: Acute immobilization stress following contextual fear conditioning reduces fear memory: timing is essential
Source: Behav Brain Funct. 2016 Feb 24;12:8. doi: 10.1186/s12993-016-0092-1 (PMC4765063; doi:10.1186/s12993-016-0092-1)
Supplement: Supplementary file 2 — 10.1186/s12993-016-0092-1 Tukey HSD for acetylation of H4K5 (Experiment 1). [file 12993_2016_92_MOESM2_ESM.docx]

Additional file 2

Table S2. Tukey HSD for acetylation of H4K5 (Experiment 1)

|  | | |  |  |  | |  |
| --- | --- | --- | --- | --- | --- | --- | --- |
|  |  | Mean difference  (I-J) |  |  | 95% Confidence  Interval | | |
|  |  |  |  |  |  |  |  |
| (I) Course | (J) Course |  | Std. Error | Sig. | Lower Bound | Upper Bound | |
| no training | 0' | -0.41818182 | 0.273272113 | 0.651758099 | -1.30603421 | 0.469670575 | |
|  | 30' | 0.378787878 | 0.292140177 | 0.782479904 | -0.57036625 | 1.327942012 | |
|  | 60' | -0.03636364 | 0.273272113 | 0.999992741 | -0.92421603 | 0.851488757 | |
|  | 90' | 0.006818182 | 0.273272113 | 0.999999998 | -0.88103421 | 0.894670575 | |
|  | 120' | 0.309090909 | 0.292140177 | 0.890281626 | -0.64006322 | 1.258245042 | |
| 0' | no training | 0.418181819 | 0.273272113 | 0.651758099 | -0.46967057 | 1.306034212 | |
|  | 30' | 0.796969697 | 0.273272113 | 0.091336141 | -0.0908827 | 1.68482209 | |
|  | 60' | 0.381818182 | 0.253000815 | 0.66411377 | -0.44017341 | 1.203809773 | |
|  | 90' | 0.425 | 0.253000815 | 0.563606807 | -0.39699159 | 1.246991592 | |
|  | 120' | 0.727272727 | 0.273272113 | 0.141907701 | -0.16057967 | 1.615125121 | |
| 30' | no training | -0.37878788 | 0.292140177 | 0.782479904 | -1.32794201 | 0.570366255 | |
|  | 0' | -0.7969697 | 0.273272113 | 0.091336141 | -1.68482209 | 0.090882697 | |
|  | 60' | -0.41515152 | 0.273272113 | 0.658253059 | -1.30300391 | 0.472700878 | |
|  | 90' | -0.3719697 | 0.273272113 | 0.748084851 | -1.25982209 | 0.515882697 | |
|  | 120' | -0.06969697 | 0.292140177 | 0.999869361 | -1.0188511 | 0.879457164 | |
| 60' | no training | 0.036363637 | 0.273272113 | 0.999992741 | -0.85148876 | 0.92421603 | |
|  | 0' | -0.38181818 | 0.253000815 | 0.66411377 | -1.20380977 | 0.44017341 | |
|  | 30' | 0.415151515 | 0.273272113 | 0.658253059 | -0.47270088 | 1.303003909 | |
|  | 90' | 0.043181818 | 0.253000815 | 0.999975012 | -0.77880977 | 0.86517341 | |
|  | 120' | 0.345454546 | 0.273272113 | 0.799062659 | -0.54239785 | 1.233306939 | |
| 90' | no training | -0.00681818 | 0.273272113 | 0.999999998 | -0.89467058 | 0.881034212 | |
|  | 0' | -0.425 | 0.253000815 | 0.563606807 | -1.24699159 | 0.396991591 | |
|  | 30' | 0.371969697 | 0.273272113 | 0.748084851 | -0.5158827 | 1.25982209 | |
|  | 60' | -0.04318182 | 0.253000815 | 0.999975012 | -0.86517341 | 0.778809773 | |
|  | 120' | 0.302272727 | 0.273272113 | 0.871425219 | -0.58557967 | 1.19012512 | |
| 120' | no training | -0.30909091 | 0.292140177 | 0.890281626 | -1.25824504 | 0.640063225 | |
|  | 0' | -0.72727273 | 0.273272113 | 0.141907701 | -1.61512512 | 0.160579666 | |
|  | 30' | 0.06969697 | 0.292140177 | 0.999869361 | -0.87945716 | 1.018851103 | |
|  | 60' | -0.34545455 | 0.273272113 | 0.799062659 | -1.23330694 | 0.542397848 | |
|  | 90' | -0.30227273 | 0.273272113 | 0.871425219 | -1.19012512 | 0.585579666 | |
| * The mean difference is significant at the 0.05 level. | | | |  |  |  | |
