# Supplementary material for: Acute immobilization stress following contextual fear conditioning reduces fear memory: timing is essential
Source: Behav Brain Funct. 2016 Feb 24;12:8. doi: 10.1186/s12993-016-0092-1 (PMC4765063; doi:10.1186/s12993-016-0092-1)
Supplement: Supplementary file 3 — 10.1186/s12993-016-0092-1 Tukey HSD for behavioral test 1 (Experiment 2). [file 12993_2016_92_MOESM3_ESM.docx]

Additional file 3

Table S3. Tukey HSD for behavioral test 1 (Experiment 2)

|  | |  |  |  |  |  |
| --- | --- | --- | --- | --- | --- | --- |
|  |  | Mean difference (I-J) |  |  | 95% Confidence  Interval | |
|  |  |  |  |  |  |  |
| (I) Course | (J) Course |  | Std. Error | Sig. | Lower Bound | Upper Bound |
| no training | training | -44.64444* | 6.9260675 | 6.63E-07 | -64.324519 | -24.96437 |
|  | training + stress (60-90') | -17.222222 | 6.9260675 | 0.1118505 | -36.902296 | 2.457852 |
|  | training + stress (90-120') | -38.83333* | 6.9260675 | 1.148E-05 | -58.513408 | -19.153259 |
|  | immobilization only | 0.67 | 6.9260675 | 0.9999794 | -19.010074 | 20.350074 |
| training | no training | 44.64444* | 6.9260675 | 6.63E-07 | 24.96437 | 64.324519 |
|  | training + stress (60-90') | 27.42222* | 6.9260675 | 0.0023481 | 7.742148 | 47.102296 |
|  | training + stress (90-120') | 5.8111111 | 6.9260675 | 0.9169482 | -13.868963 | 25.491185 |
|  | immobilization only | 45.31444* | 6.9260675 | 4.766E-07 | 25.63437 | 64.994519 |
| training + stress (60-90') | no training | 17.222222 | 6.9260675 | 0.1118505 | -2.457852 | 36.902296 |
|  | training | -27.42222* | 6.9260675 | 0.0023481 | -47.102296 | -7.742148 |
|  | training + stress (90-120') | -21.61111* | 6.9260675 | 0.0249108 | -41.291185 | -1.9310369 |
|  | immobilization only | 17.892222 | 6.9260675 | 0.0907004 | -1.787852 | 37.572296 |
| training + stress (90-120') | no training | 38.83333* | 6.9260675 | 1.148E-05 | 19.153259 | 58.513408 |
|  | training | -5.8111111 | 6.9260675 | 0.9169482 | -25.491185 | 13.868963 |
|  | training + stress (60-90') | 21.61111* | 6.9260675 | 0.0249108 | 1.9310369 | 41.291185 |
|  | immobilization only | 39.50333* | 6.9260675 | 8.283E-06 | 19.823259 | 59.183408 |
| immobilization only | no training | -0.67 | 6.9260675 | 0.9999794 | -20.350074 | 19.010074 |
|  | training | -45.31444* | 6.9260675 | 4.766E-07 | -64.994519 | -25.63437 |
|  | training + stress (60-90') | -17.892222 | 6.9260675 | 0.0907004 | -37.572296 | 1.787852 |
|  | training + stress (90-120') | -39.50333* | 6.9260675 | 8.283E-06 | -59.183408 | -19.823259 |
| * The mean difference is significant at the 0.05 level. | | | |  |  |  |
