# Supplementary material for: Acute immobilization stress following contextual fear conditioning reduces fear memory: timing is essential
Source: Behav Brain Funct. 2016 Feb 24;12:8. doi: 10.1186/s12993-016-0092-1 (PMC4765063; doi:10.1186/s12993-016-0092-1)
Supplement: Supplementary file 4 — 10.1186/s12993-016-0092-1 Tukey HSD for BDNF analysis 1 (Experiment 2). [file 12993_2016_92_MOESM4_ESM.docx]

Additional file 4

Table S4. Tukey HSD for BDNF analysis 1 (Experiment 2)

|  | |  |  |  |  |  |
| --- | --- | --- | --- | --- | --- | --- |
|  |  | Mean difference (I-J) | Std.Error | Sig. | 95% Confidence  Interval | |
| (I) Course | (J) Course |  |  |  | Lower Bound | Upper Bound |
| no training | training | -1.16657^*^ | .23214 | .000 | -1.8672 | -.4659 |
|  | training + stress (60-90') | .02272 | .22534 | 1.000 | -.6574 | .7029 |
|  | training + stress (90-120') | -.70422 | .24090 | .062 | -1.4313 | .0229 |
|  | naïve | .22718 | .22534 | .912 | -.4530 | .9073 |
|  | immobilization stress only | .24049 | .25266 | .930 | -.5221 | 1.0031 |
| training | no training | 1.16657^*^ | .23214 | .000 | .4659 | 1.8672 |
|  | training + stress (60-90') | 1.18929^*^ | .21595 | .000 | .5375 | 1.8411 |
|  | training + stress (90-120') | .46235 | .23214 | .368 | -.2383 | 1.1630 |
|  | naïve | 1.39375^*^ | .21595 | .000 | .7420 | 2.0455 |
|  | immobilization stress only | 1.40706^*^ | .24432 | .000 | .6696 | 2.1445 |
| training + stress (60-90') | no training | -.02272 | .22534 | 1.000 | -.7029 | .6574 |
|  | training | -1.18929^*^ | .21595 | .000 | -1.8411 | -.5375 |
|  | training + stress (90-120') | -.72694^*^ | .22534 | .030 | -1.4071 | -.0468 |
|  | naïve | .20447 | .20863 | .921 | -.4252 | .8342 |
|  | immobilization stress only | .21777 | .23787 | .940 | -.5002 | .9357 |
| training + stress (90-120') | no training | .70422 | .24090 | .062 | -.0229 | 1.4313 |
|  | training | -.46235 | .23214 | .368 | -1.1630 | .2383 |
|  | training + stress (60-90') | .72694^*^ | .22534 | .030 | .0468 | 1.4071 |
|  | naïve | .93140^*^ | .22534 | .003 | .2513 | 1.6115 |
|  | immobilization stress only | .94471^*^ | .25266 | .008 | .1821 | 1.7073 |
| naïve | no training | -.22718 | .22534 | .912 | -.9073 | .4530 |
|  | training | -1.39375^*^ | .21595 | .000 | -2.0455 | -.7420 |
|  | training + stress (60-90') | -.20447 | .20863 | .921 | -.8342 | .4252 |
|  | training + stress (90-120') | -.93140^*^ | .22534 | .003 | -1.6115 | -.2513 |
|  | immobilization stress only | .01330 | .23787 | 1.000 | -.7047 | .7313 |
| immobilization stress only | no training | -.24049 | .25266 | .930 | -1.0031 | .5221 |
|  | training | -1.40706^*^ | .24432 | .000 | -2.1445 | -.6696 |
|  | training + stress (60-90') | -.21777 | .23787 | .940 | -.9357 | .5002 |
|  | training + stress (90-120') | -.94471^*^ | .25266 | .008 | -1.7073 | -.1821 |
|  | naïve | -.01330 | .23787 | 1.000 | -.7313 | .7047 |
| * The mean difference is significant at the 0.05 level. | |  |  |  |  |  |
