# Supplementary material for: Acute immobilization stress following contextual fear conditioning reduces fear memory: timing is essential
Source: Behav Brain Funct. 2016 Feb 24;12:8. doi: 10.1186/s12993-016-0092-1 (PMC4765063; doi:10.1186/s12993-016-0092-1)
Supplement: Supplementary file 5 — 10.1186/s12993-016-0092-1 Tukey HSD for acetylation H3K14 (Experiment 3). [file 12993_2016_92_MOESM5_ESM.docx]

|  | |  |  |  | 95% Confidence  Interval | |
| --- | --- | --- | --- | --- | --- | --- |
|  |  | Mean difference (I-J) | Std.Error | Sig. |  |  |
| (I) Course | (J) Course |  |  |  | Lower Bound | Upper Bound |
| no training | training 90' | -1.87850^*^ | .27912 | .000 | -2.7689 | -.9881 |
|  | training 120' | -.99817^*^ | .27912 | .021 | -1.8886 | -.1078 |
|  | training + stress (60-90') | .22784 | .27912 | .981 | -.6626 | 1.1182 |
|  | training + stress (90-120') | -.49075 | .29605 | .648 | -1.4352 | .4537 |
|  | naïve | .65678 | .29605 | .320 | -.2876 | 1.6012 |
|  | immobilization stress only | .36705 | .27912 | .839 | -.5234 | 1.2575 |
| training 90' | no training | 1.87850^*^ | .27912 | .000 | .9881 | 2.7689 |
|  | training 120' | .88033 | .27912 | .054 | -.0101 | 1.7707 |
|  | training + stress (60-90') | 2.10634^*^ | .27912 | .000 | 1.2159 | 2.9967 |
|  | training + stress (90-120') | 1.38775^*^ | .29605 | .001 | .4433 | 2.3322 |
|  | naïve | 2.53528^*^ | .29605 | .000 | 1.5909 | 3.4797 |
|  | immobilization stress only | 2.24555^*^ | .27912 | .000 | 1.3551 | 3.1360 |
| training 120' | no training | .99817^*^ | .27912 | .021 | .1078 | 1.8886 |
|  | training 90' | -.88033 | .27912 | .054 | -1.7707 | .0101 |
|  | training + stress (60-90') | 1.22601^*^ | .27912 | .003 | .3356 | 2.1164 |
|  | training + stress (90-120') | .50742 | .29605 | .613 | -.4370 | 1.4518 |
|  | naïve | 1.65495^*^ | .29605 | .000 | .7105 | 2.5994 |
|  | immobilization stress only | 1.36522^*^ | .27912 | .001 | .4748 | 2.2556 |
| training + stress (60-90') | no training | -.22784 | .27912 | .981 | -1.1182 | .6626 |
|  | training 90' | -2.10634^*^ | .27912 | .000 | -2.9967 | -1.2159 |
|  | training 120' | -1.22601^*^ | .27912 | .003 | -2.1164 | -.3356 |
|  | training + stress (90-120') | -.71859 | .29605 | .228 | -1.6630 | .2258 |
|  | naïve | .42894 | .29605 | .771 | -.5155 | 1.3734 |
|  | immobilization stress only | .13921 | .27912 | .999 | -.7512 | 1.0296 |
| training + stress (90-120') | no training | .49075 | .29605 | .648 | -.4537 | 1.4352 |
|  | training 90' | -1.38775^*^ | .29605 | .001 | -2.3322 | -.4433 |
|  | training 120' | -.50742 | .29605 | .613 | -1.4518 | .4370 |
|  | training + stress (60-90') | .71859 | .29605 | .228 | -.2258 | 1.6630 |
|  | naïve | 1.14753^*^ | .31206 | .016 | .1520 | 2.1430 |
|  | immobilization stress only | .85780 | .29605 | .093 | -.0866 | 1.8022 |
| naïve | no training | -.65678 | .29605 | .320 | -1.6012 | .2876 |
|  | training 90' | -2.53528^*^ | .29605 | .000 | -3.4797 | -1.5909 |
|  | training 120' | -1.65495^*^ | .29605 | .000 | -2.5994 | -.7105 |
|  | training + stress (60-90') | -.42894 | .29605 | .771 | -1.3734 | .5155 |
|  | training + stress (90-120') | -1.14753^*^ | .31206 | .016 | -2.1430 | -.1520 |
|  | immobilization stress only | -.28973 | .29605 | .954 | -1.2341 | .6547 |
| immobilization stress only | no training | -.36705 | .27912 | .839 | -1.2575 | .5234 |
|  | training 90' | -2.24555^*^ | .27912 | .000 | -3.1360 | -1.3551 |
|  | training 120' | -1.36522^*^ | .27912 | .001 | -2.2556 | -.4748 |
|  | training + stress (60-90') | -.13921 | .27912 | .999 | -1.0296 | .7512 |
|  | training + stress (90-120') | -.85780 | .29605 | .093 | -1.8022 | .0866 |
|  | naïve | .28973 | .29605 | .954 | -.6547 | 1.2341 |
| * The mean difference is significant at the 0.05 level. | |  |  |  |  |  |

Additional file 5

Table S5. Tukey HSD for acetylation H3K14 (Experiment 3)
