# Supplementary material for: Acute immobilization stress following contextual fear conditioning reduces fear memory: timing is essential
Source: Behav Brain Funct. 2016 Feb 24;12:8. doi: 10.1186/s12993-016-0092-1 (PMC4765063; doi:10.1186/s12993-016-0092-1)
Supplement: Supplementary file 6 — 10.1186/s12993-016-0092-1 Tukey HSD for acetylation H3K14 at promoter 3 (Experiment 4). [file 12993_2016_92_MOESM6_ESM.docx]

Additional file 6

Table S6. Tukey HSD for acetylation H3K14 at promoter 3 (Experiment 4)

|  | | |  |  |  |  |
| --- | --- | --- | --- | --- | --- | --- |
|  |  | Mean difference  (I-J) | Std.Error | Sig. | 95% Confidence  Interval | |
| (I) Course | (J) Course |  |  |  | Lower Bound | Upper Bound |
| no training | training 90' | -1.13523^*^ | .19292 | .001 | -1.6865 | -.5840 |
|  | training + stress (60-90') | -.12162 | .19292 | .808 | -.6729 | .4296 |
| training 90' | no training | 1.13523^*^ | .19292 | .001 | .5840 | 1.6865 |
|  | training + stress (60-90') | 1.01361^*^ | .17861 | .001 | .5032 | 1.5240 |
| training + stress (60-90') | no training | .12162 | .19292 | .808 | -.4296 | .6729 |
|  | training 90' | -1.01361^*^ | .17861 | .001 | -1.5240 | -.5032 |
| * The mean difference is significant at the 0.05 level. | | |  |  |  |  |
