# Supplementary material for: Acute immobilization stress following contextual fear conditioning reduces fear memory: timing is essential
Source: Behav Brain Funct. 2016 Feb 24;12:8. doi: 10.1186/s12993-016-0092-1 (PMC4765063; doi:10.1186/s12993-016-0092-1)
Supplement: Supplementary file 7 — 10.1186/s12993-016-0092-1 Tukey HSD for acetylation H3K14 at promoter 4 (Experiment 4). [file 12993_2016_92_MOESM7_ESM.docx]

Additional file 7

Table S7. Tukey HSD for acetylation H3K14 at promoter 4 (Experiment 4)

|  | | |  |  |  |  |
| --- | --- | --- | --- | --- | --- | --- |
|  |  | Mean difference (I-J) | Std.Error | Sig. | 95% Confidence  Interval | |
| (I) Course | (J) Course |  |  |  | Lower Bound | Upper Bound |
| no training | training 90' | -.06700 | .40425 | .985 | -1.1957 | 1.0617 |
|  | training + stress (60-90') | -.09442 | .40425 | .970 | -1.2231 | 1.0342 |
| training 90' | no training | .06700 | .40425 | .985 | -1.0617 | 1.1957 |
|  | training + stress (60-90') | -.02741 | .40425 | .997 | -1.1561 | 1.1012 |
| training + stress (60-90') | no training | .09442 | .40425 | .970 | -1.0342 | 1.2231 |
|  | training 90' | .02741 | .40425 | .997 | -1.1012 | 1.1561 |
| * The mean difference is significant at the 0.05 level. | | |  |  |  |  |
