# Supplementary material for: Acute immobilization stress following contextual fear conditioning reduces fear memory: timing is essential
Source: Behav Brain Funct. 2016 Feb 24;12:8. doi: 10.1186/s12993-016-0092-1 (PMC4765063; doi:10.1186/s12993-016-0092-1)
Supplement: Supplementary file 8 — 10.1186/s12993-016-0092-1 Tukey HSD for corticosterone analysis (Experiment 5). [file 12993_2016_92_MOESM8_ESM.docx]

Additional file 8

Table S8. Tukey HSD for corticosterone analysis (Experiment 5)

|  | |  |  |  |  |  |
| --- | --- | --- | --- | --- | --- | --- |
|  |  | Mean difference  (I-J) | Std.Error | Sig. | 95% Confidence  Interval | |
| (I) Course | (J) Course |  |  |  | Lower Bound | Upper Bound |
| no training | training | -137.66329 | 81.45374 | .462 | -381.4034 | 106.0768 |
|  | training + stress (60-90') | -506.14051^*^ | 81.45374 | .000 | -749.8806 | -262.4004 |
|  | training + stress (90-120') | -161.48479 | 81.45374 | .310 | -405.2249 | 82.2553 |
|  | immobilization stress only | -298.79052^*^ | 81.45374 | .012 | -542.5306 | -55.0504 |
| training | no training | 137.66329 | 81.45374 | .462 | -106.0768 | 381.4034 |
|  | training + stress (60-90') | -368.47722^*^ | 81.45374 | .002 | -612.2173 | -124.7371 |
|  | training + stress (90-120') | -23.82150 | 81.45374 | .998 | -267.5616 | 219.9186 |
|  | immobilization stress only | -161.12723 | 81.45374 | .312 | -404.8673 | 82.6129 |
| training + stress (60-90') | no training | 506.14051^*^ | 81.45374 | .000 | 262.4004 | 749.8806 |
|  | training | 368.47722^*^ | 81.45374 | .002 | 124.7371 | 612.2173 |
|  | training + stress (90-120') | 344.65572^*^ | 81.45374 | .003 | 100.9156 | 588.3958 |
|  | immobilization stress only | 207.34999 | 81.45374 | .120 | -36.3901 | 451.0901 |
| training + stress (90-120′) | no training | 161.48479 | 81.45374 | .310 | -82.2553 | 405.2249 |
|  | training | 23.82150 | 81.45374 | .998 | -219.9186 | 267.5616 |
|  | training + stress (60-90') | -344.65572^*^ | 81.45374 | .003 | -588.3958 | -100.9156 |
|  | immobilization stress only | -137.30573 | 81.45374 | .464 | -381.0458 | 106.4344 |
| immobilization stress only | no training | 298.79052^*^ | 81.45374 | .012 | 55.0504 | 542.5306 |
|  | training | 161.12723 | 81.45374 | .312 | -82.6129 | 404.8673 |
|  | training + stress (60-90') | -207.34999 | 81.45374 | .120 | -451.0901 | 36.3901 |
|  | training + stress (90-120') | 137.30573 | 81.45374 | .464 | -106.4344 | 381.0458 |
| * The mean difference is significant at the 0.05 level. | |  |  |  |  |  |
