# Supplementary material for: Acute immobilization stress following contextual fear conditioning reduces fear memory: timing is essential
Source: Behav Brain Funct. 2016 Feb 24;12:8. doi: 10.1186/s12993-016-0092-1 (PMC4765063; doi:10.1186/s12993-016-0092-1)
Supplement: Supplementary file 9 — 10.1186/s12993-016-0092-1 Tukey HSD for behavioral test 2 (Experiment 6). [file 12993_2016_92_MOESM9_ESM.docx]

Additional file 9

Table S9. Tukey HSD for behavioral test 2 (Experiment 6)

|  | |  |  |  |  |  |
| --- | --- | --- | --- | --- | --- | --- |
|  |  | Mean difference  (I-J) |  |  |  |  |
|  |  |  |  |  | 95% Confidence  Interval | |
| (I) Course | (J) Course |  | Std.Error | Sig. | Lower Bound | Upper Bound |
| no training | training (veh) | -48.88889* | 5.554597883 | 6.61616E-11 | -65.28903 | -32.4887477 |
|  | training (mif) | -45.32922* | 5.413952324 | 3.22727E-10 | -61.3141025 | -29.3443419 |
|  | training (veh) + stress | -22.58586* | 5.29610128 | 0.001071029 | -38.2227796 | -6.94893755 |
|  | training (mif) + stress | -48.40404* | 5.29610128 | 1.94917E-11 | -64.0409614 | -32.7671194 |
|  | mif only | 0.777777778 | 5.29610128 | 0.999989909 | -14.8591433 | 16.41469881 |
| training (veh) | no training | 48.88889* | 5.554597883 | 6.61616E-11 | 32.48874775 | 65.28903003 |
|  | training (mif) | 3.559666667 | 5.413952324 | 0.985770447 | -12.4252136 | 19.54454696 |
|  | training (veh) + stress | 26.30303* | 5.29610128 | 9.85955E-05 | 10.66610927 | 41.93995134 |
|  | training (mif) + stress | 0.484848485 | 5.29610128 | 0.999999042 | -15.1520726 | 16.12176952 |
|  | mif only | 49.66667* | 5.29610128 | 8.50686E-12 | 34.02974563 | 65.3035877 |
| training (mif) | no training | 45.32922* | 5.413952324 | 3.22727E-10 | 29.34434193 | 61.31410251 |
|  | training (veh) | -3.55966667 | 5.413952324 | 0.985770447 | -19.544547 | 12.42521362 |
|  | training (veh) + stress | 22.74336* | 5.148398866 | 0.000646239 | 7.542539054 | 37.94418822 |
|  | training (mif) + stress | -3.07481818 | 5.148398866 | 0.990804031 | -18.2756428 | 12.1260064 |
|  | mif only | 46.10700* | 5.148398866 | 3.77274E-11 | 30.90617542 | 61.30782458 |
| training (veh) + stress | no training | 22.58586* | 5.29610128 | 0.001071029 | 6.948937551 | 38.22277962 |
|  | training (veh) | -26.30303* | 5.29610128 | 9.85955E-05 | -41.9399513 | -10.6661093 |
|  | training (mif) | -22.74336* | 5.148398866 | 0.000646239 | -37.9441882 | -7.54253905 |
|  | training (mif) + stress | -25.81818* | 5.024322829 | 5.36991E-05 | -40.6526676 | -10.983696 |
|  | mif only | 23.36364* | 5.024322829 | 0.000294852 | 8.529150545 | 38.19812218 |
| training (mif) + stress | no training | 48.40404* | 5.29610128 | 1.94917E-11 | 32.76711937 | 64.04096144 |
|  | training (veh) | -0.48484848 | 5.29610128 | 0.999999042 | -16.1217695 | 15.15207255 |
|  | training (mif) | 3.074818182 | 5.148398866 | 0.990804031 | -12.1260064 | 18.27564276 |
|  | training (veh) + stress | 25.81818* | 5.024322829 | 5.36991E-05 | 10.983696 | 40.65266764 |
|  | mif only | 49.18182* | 5.024322829 | 2.37976E-12 | 34.34733236 | 64.016304 |
| mif only | no training | -0.77777778 | 5.29610128 | 0.999989909 | -16.4146988 | 14.85914326 |
|  | training (veh) | -49.66667* | 5.29610128 | 8.50686E-12 | -65.3035877 | -34.0297456 |
|  | training (mif) | -46.10700* | 5.148398866 | 3.77274E-11 | -61.3078246 | -30.9061754 |
|  | training (veh) + stress | -23.36364* | 5.024322829 | 0.000294852 | -38.1981222 | -8.52915054 |
|  | training (mif) + stress | -49.18182* | 5.024322829 | 2.37976E-12 | -64.016304 | -34.3473324 |
| * The mean difference is significant at the 0.05 level. | | |  |  |  |  |
