# Supplementary material for: Acute immobilization stress following contextual fear conditioning reduces fear memory: timing is essential
Source: Behav Brain Funct. 2016 Feb 24;12:8. doi: 10.1186/s12993-016-0092-1 (PMC4765063; doi:10.1186/s12993-016-0092-1)
Supplement: Supplementary file 10 — 10.1186/s12993-016-0092-1 Tukey HSD for mRNA 2 (Experiment 6). [file 12993_2016_92_MOESM10_ESM.docx]

Additional file 10

Table S10. Tukey HSD for mRNA 2 (Experiment 6)

|  | | Mean difference  (I-J) |  |  |  |  |
| --- | --- | --- | --- | --- | --- | --- |
|  |  |  |  |  | 95% Confidence  Interval | |
| (I) Course | (J) Course |  | Std.Error | Sig. | Lower Bound | Upper Bound |
| no training | training (veh) | -1.05238* | 0.329797771 | 0.033170691 | -2.04779208 | -0.05697303 |
|  | training (mif) | -1.03716* | 0.343264403 | 0.049625877 | -2.07321823 | -0.00110807 |
|  | training (veh) + stress | 0.275261213 | 0.343264403 | 0.965216131 | -0.76079387 | 1.311316292 |
|  | training (mif) + stress | -0.8618579 | 0.343264403 | 0.149357367 | -1.89791298 | 0.174197175 |
|  | mif only | 0.353178004 | 0.319325319 | 0.875487781 | -0.61062312 | 1.316979131 |
| training (veh) | no training | 1.05238* | 0.329797771 | 0.033170691 | 0.05697303 | 2.047792076 |
|  | training (mif) | 0.015219401 | 0.343264403 | 0.999999973 | -1.02083568 | 1.051274481 |
|  | training (veh) + stress | 1.32764* | 0.343264403 | 0.005797622 | 0.291588686 | 2.363698845 |
|  | training (mif) + stress | 0.190524649 | 0.343264403 | 0.99323642 | -0.84553043 | 1.226579728 |
|  | mif only | 1.40556* | 0.319325319 | 0.001312852 | 0.441759431 | 2.369361683 |
| training (mif) | no training | 1.03716* | 0.343264403 | 0.049625877 | 0.001108072 | 2.073218232 |
|  | training (veh) | -0.0152194 | 0.343264403 | 0.999999973 | -1.05127448 | 1.020835679 |
|  | training (veh) + stress | 1.31242* | 0.356222306 | 0.009473049 | 0.237259197 | 2.387589533 |
|  | training (mif) + stress | 0.175305248 | 0.356222306 | 0.996139927 | -0.89985992 | 1.250470416 |
|  | mif only | 1.39034* | 0.333215456 | 0.002505321 | 0.384616233 | 2.396066079 |
| training (veh) + stress | no training | -0.27526121 | 0.343264403 | 0.965216131 | -1.31131629 | 0.760793867 |
|  | training (veh) | -1.32764* | 0.343264403 | 0.005797622 | -2.36369885 | -0.29158869 |
|  | training (mif) | -1.31242* | 0.356222306 | 0.009473049 | -2.38758953 | -0.2372592 |
|  | training (mif) + stress | -1.13712* | 0.356222306 | 0.033076932 | -2.21228428 | -0.06195395 |
|  | mif only | 0.077916792 | 0.333215456 | 0.999894861 | -0.92780813 | 1.083641715 |
| training (mif) + stress | no training | 0.861857904 | 0.343264403 | 0.149357367 | -0.17419718 | 1.897912984 |
|  | training (veh) | -0.19052465 | 0.343264403 | 0.99323642 | -1.22657973 | 0.845530431 |
|  | training (mif) | -0.17530525 | 0.356222306 | 0.996139927 | -1.25047042 | 0.89985992 |
|  | training (veh) + stress | 1.13712* | 0.356222306 | 0.033076932 | 0.061953949 | 2.212284285 |
|  | mif only | 1.21504* | 0.333215456 | 0.010468567 | 0.209310986 | 2.220760832 |
| mif only | no training | -0.353178 | 0.319325319 | 0.875487781 | -1.31697913 | 0.610623122 |
|  | training (veh) | -1.40556* | 0.319325319 | 0.001312852 | -2.36936168 | -0.44175943 |
|  | training (mif) | -1.39034* | 0.333215456 | 0.002505321 | -2.39606608 | -0.38461623 |
|  | training (veh) + stress | -0.07791679 | 0.333215456 | 0.999894861 | -1.08364171 | 0.927808131 |
|  | training (mif) + stress | -1.21504* | 0.333215456 | 0.010468567 | -2.22076083 | -0.20931099 |
| * The mean difference is significant at the 0.05 level. | | |  |  |  |  |
